# Wound Induced Tanscriptional Regulation of Benzylisoquinoline Pathway and Characterization of Wound Inducible PsWRKY Transcription Factor from *Papaver somniferum*

**DOI:** 10.1371/journal.pone.0052784

**Published:** 2013-01-30

**Authors:** Sonal Mishra, Vineeta Triptahi, Seema Singh, Ujjal J. Phukan, M. M. Gupta, Karuna Shanker, Rakesh Kumar Shukla

**Affiliations:** 1 Biotechnology Division, Central Institute of Medicinal and Aromatic Plants (CSIR-CIMAP), Lucknow, India; 2 Analytical Chemistry Division, Central Institute of Medicinal and Aromatic Plants (CSIR-CIMAP), Lucknow, India; 3 Botany Department, CSIR-Central Drug Research Institute (CSIR-CDRI), Lucknow, India; RIKEN Plant Science Center, Japan

## Abstract

Wounding is required to be made in the walls of the green seed pod of Opium poppy prior exudation of latex. To withstand this kind of trauma plants regulate expression of some metabolites through an induced transcript level. 167 unique wound-inducible ESTs were identified by a repetitive round of cDNA subtraction after 5 hours of wounding in *Papaver somniferum* seedlings. Further repetitive reverse northern analysis of these ESTs revealed 80 transcripts showing more than two fold induction, validated through semi-quantitative RT-PCR & real time expression analysis. One of the major classified categories among identified ESTs belonged to benzylisoquinoline transcripts. Tissue specific metabolite analysis of benzylisoquinoline alkaloids (BIAs) in response to wounding revealed increased accumulation of narcotine and papaverine. Promoter analysis of seven transcripts of BIAs pathway showed the presence of W-box *cis*-element with the consensus sequence of TGAC, which is the proposed binding site for WRKY type transcription factors. One of the Wound inducible ‘WRKY’ EST isolated from our subtracted library was made full-length and named as ‘*PsWRKY’*. Bacterially expressed PsWRKY interacted with the W-box element having consensus sequence TTGACT/C present in the promoter region of BIAs biosynthetic pathway genes. PsWRKY further activated the TYDC promoter in yeast and transiently in tobacco BY2 cells. Preferential expression of *PsWRKY* in straw and capsule and its interaction with consensus W-box element present in BIAs pathway gene transcripts suggest its possible involvement in the wound induced regulation of BIAs pathway.

## Introduction

**Figure 1:**
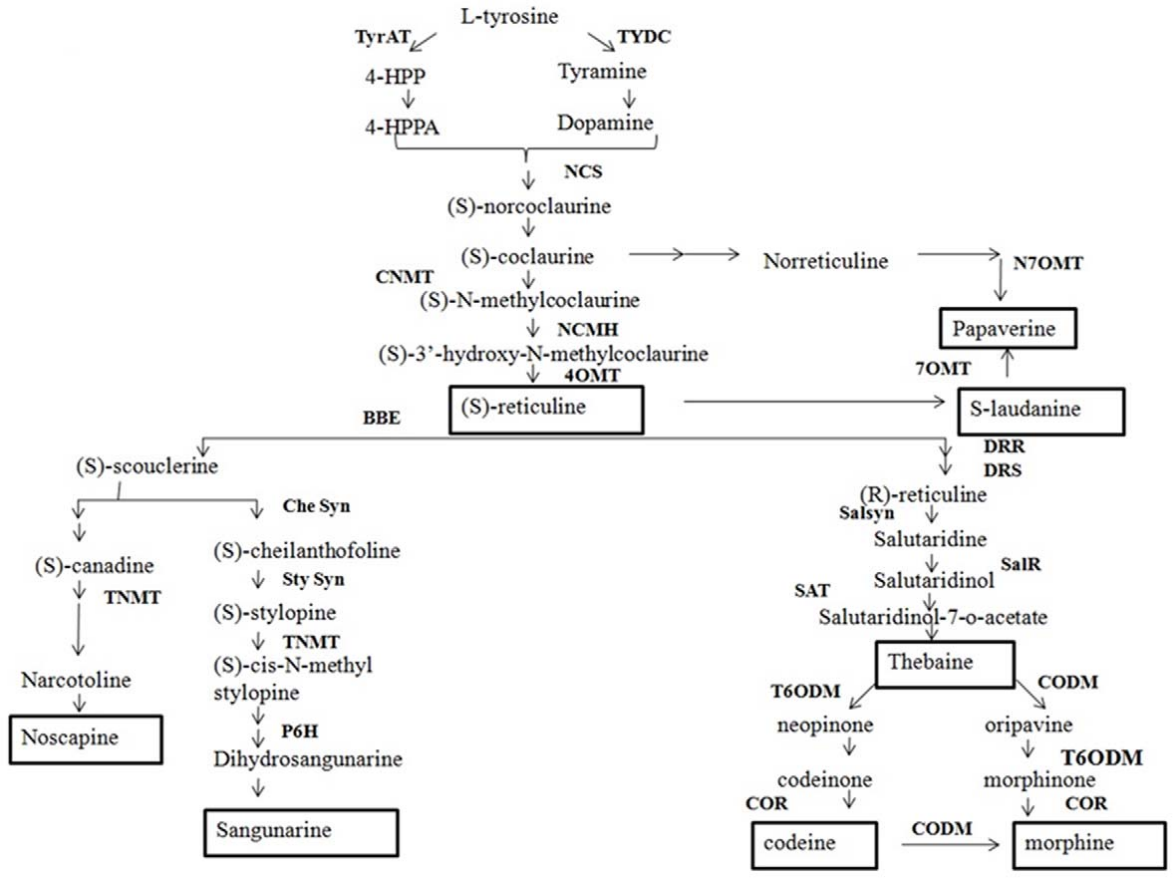
Schematic representation of BIA pathway. Biosynthetic pathway and corresponding enzymes leading to synthesis of **benzylisoquinolines** (papaverine, reticuline, laudanine), **morphinans** (thebaine, oripavine, codeine, morphine), **protoberberines** (berberine), **phthalideisoquinolines** (narcotoline, noscapine), **benzophenanthridines** (sanguinarine) and **papaverrubine.** Abbreviations: **TYDC** tyrosine/dopa decarboxylase; **TyrAT** tyrosine aminotransferase; **NCS** norcoclaurine synthase; **6OMT** (S)-norcoclaurine 6-O-methyltransferase; **CNMT** (S)-coclaurine Nmethyltransferase; **NMCH** (S)-N-methylcoclaurine 3-hydroxylase; **4-OMT** (S)-30-hydroxy-N-methylcoclaurine 4-O-methyltransferase; **N7OMT** norreticuline 7-O-methyltransferase; **7OMT** reticuline 7-Omethyltransferase; **BBE** berberine bridge enzyme; **SOMT** scoulerine 9-O-methyltransferase; **CAS** canadine synthase; **STOX** (S)-tetrahydroxyprotoberberine oxidase; **CoOMT** Columbamine O-methyltransferase; **CheSyn** cheilanthifoline synthase; **StySyn** stylopine synthase; **TNMT** tetrahydroprotoberberine N-methyltransferase; **MSH** methylstylopine hydroxylase; **P6H** protopine 6-hydroxylase; **DBOX** dihydrobenzophenanthridine oxidase; **DRS** 1,2-dehydroreticuline synthase; **DRR** 1,2-dehydroreticuline reductase; **SalSyn** salutaridine synthase; **SalR** salutaridine reductase; **SalAT** salutaridinol 7-O-acetyltransferase; **T6ODM** thebaine 6-O-demethylase; **COR** codeinone reductase; **CODM** codeine O-demethylase.

*Papaver somniferum* is an important medicinal plant. Its medicinal properties are due to benzylisoquinoline alkaloids (BIAs) [1]. BIAs represents approximately 25, 00 elucidated natural product structures found mainly in Papaveraceae, Ranunculaceae, Berberidaceae and Menispermaceae [2]. BIA biosynthesis start with the formation of two L-tyrosine derivatives, 4-hydroxyphenylacetaldehyde (4-HPAA) and dopamine (Figure 1). The synthesis of 4-HPAA and dopamine from tyrosine is catalyzed by tyrosine aminotransferase (TyrAT) and tyrosine/DOPA decarboxylase (TYDC) respectively [3], [4]. Norcoclaurine synthase (NCS) catalyzes the condensation of 4-HPAA and dopamine yielding (S)-norcoclaurine [5]. Subsequently (S)-methylcoclaurine is synthesized after sequential methylations by norcoclaurine 6-O-methyltransferase (6OMT) and coclaurine N-methyltransferase (CNMT) [6]. (S)-N-Methylcoclaurine-3-hydroxylase (NMCH) catalyzes the 3-hydroxylation of (S)-N-methylcoclaurine to (S)-3-hydroxy-N-methylcoclaurine, which is further converted by 3-hydroxy-N-methylcoclaurine 4-O-methyltransferase (4OMT) to (S)-reticuline [6], [7]. (S)-reticuline, is the central intermediate from which morphinan alkaloid branch pathway starts with the epimerization of (S)-reticuline to (R)-reticuline by 1,2-dehydroreticuline synthase (DRS) and 1,2-dehydroreticuline reductase (DRR) [8], [9]. (R)-reticuline is converted to salutaridine by salutaridine synthase (SalSyn) which is subsequently reduced by salutaridine reductase [9], [10]. Salutaridinol 7-O-acetyltransferase (SalAT) catalyzes the conversion of resulting intermediate to salutaridinol-7-O-acetate [11]; Salutaridinol-7-O-acetate is further rearranges itself spontaneously or enzymatically to thebaine. Thebaine 6-O- demethylase (T6ODM) and Codeine O-demethylase (CODM) catalyze demethylation of thebaine to oripavine and neopinone [12]. T6ODM further catalyzes conversion of oripavine to morphinone [12]. The NADPH dependent enzyme codeinone reductase (COR) converts (2)-codeinone to (2)-codeine as the penultimate step in the morphine biosynthetic pathway [13]. Most of the enzymes involved in morphinone biosynthetic pathway are characterized, but not much information is known for the branched pathway which leads to formation of noscapine, sanguinarine and papaverine. The Non-Morphinan alkaloid biosynthesis begins with the conversion of (S)-reticuline to (S)-scoulerine, the first committed step catalyzed by berberine bridge enzyme (BBE) [6], [14], [15], [16]. From here the pathway gets diverted for sanguinarine alkaloid biosynthesis initiated by the synthesis of (S)-stylopine, catalyzed by CYPs cheilanthifoline synthase (CheSyn) and stylopine synthase (STSY) [17], [18]. The subsequent step for the dihydrosanguinarine synthesis involves tetrahydroprotoberberine *cis*-N-methyltransferase (TNMT), methylstylopine 14-hydroxylase (MSH) and protopine 6-hydroxylase (P6H) [19], [20], [21]. Dihydrosanguinarine is oxidized to sanguinarine by dihydrobenzophenanthridine oxidase (DBOX) [22], [23]. The second diversion from (S)-scoulerine is turned on by Scoulerine 9-O-methyltransferase (SOMT) [24], [25], which converts (S)-scoulerine to (S)-tetrahydrocolumbamine. In next step canadinesynthase forms (S)-canadine [26] and subsequently, tetrahydroprotoberberine N-methyltransferase (TNMT) yields N-methylcanadine [19]. The formation of narcotoline occurs, which is O-methylated to noscapine [16]. Palmatine biosynthesis is reported in Coptis japonica, proceeds via columbamine or tetrahydropalmatine involving (S)-tetrahydroxyprotoberberine oxidase (STOX) and columbamine O-methyltransferase (CoOMT) [27], [28]. Biosynthesis of Papaverine is not well understood, however, two pathways have been proposed. The first one begins with the conversion of (S)-reticuline to (S)-laudanine by reticuline 7-O-methyltransferase (7OMT) [29], [30], and as proposed in second it starts at (S)-coclaurine and involve an unique 30-hydroxylase similar to NMCH, norreticuline7-O-methyltransferase (N7OMT). A generalized scheme of the BIAs pathway is shown in Figure 1.

Morphine is the major alkaloid found in the latex of highly specialized and articulated laticifer cells that are derived from phloem [2], [31]. Morphine induces cross linking of galacturonic-containing polysaccharides in the cell walls of opium poppy [15], [32]. Sanguinarine and berberine are toxic to some herbivores and microbial plant pathogens; hence they are proposed to function as defense compounds [33], [34]. Sangurine is induced in response to treatment with fungal derived elicitor in cell- culture of opium poppy [35]. In contrast, morphine does not accumulate in induced or non-induced cell cultures [34], [36]. Alkaloids of BIAs biosynthetic pathway start accumulating after seed imbibition [36]. TYDC and BBE mRNA were induced in response to methyl jasmonate and elicitor treatment [36], [37], [38], [39]. Wound induced transcripts of BIAs biosynthetic pathway studied in cell culture include *TYDC*, *CNMT*, *6-OMT*, *4-OMT*, *BBE*, and *SAT* [36]. Induction of these transcripts in cell culture was unexpected, as de-differentiated opium poppy cell- culture did not accumulate morphine or its pathway intermediates [36]. Induction of BIAs transcripts and non availability of morphinan alkaloids in cell culture in response to external signals prompted us to understand the regulation of BIAs pathway after wounding in *P. somniferum* plant.

A number of transcriptional regulators regulating TIA pathway form *C. roseus* have been identified and characterized [40]. TIA production is also increased by ectopic expression of genes encoding rate limiting enzymes. Such as overexpression of TDC (tryptophan decarboxylase) gene in *C. roseus* led to moderate increase in alkaloid accumulation [41]. A more promising approach is the ectopic expression of transcription factors that can regulate multiple steps of the pathway leading to increased accumulation of metabolites [42]. These transcriptional regulators are the site of single step manipulation of metabolic pathways and can be much more effective in metabolic engineering of multiple step biosynthetic pathways [43]. Some of the characterized AP2 like transcription factors positively regulating the TIA pathway are *ORCA3*, *ORCA2 and ORCA1. O*n the other hand ZCT1, ZCT2 and ZCT3 are the zinc finger repressors of TIA pathway [44], [45]. WRKY transcription factor families are shown to be involved in alkaloid biosynthesis. The WRKY family proteins contain one or two copies of a DNA-binding domain, designated as WRKY domain, composed of about 50–60 amino acids with the N-terminal conserved motif WRKYGXK and a zinc finger motif (C-X4-8-C-X22-28-H-X1-2-H/C) at the C-terminus [46]. WRKY proteins regulate expression of downstream genes by interacting with the *cis*-element W-box [TTGAC (T/C)], localized in the promoter regions of the target genes [47], [48]. WRKY family TFs has shown diversified role in defense, development and metabolism [47]. Recently identified CrWRKY transcription factor from *C. roseus* activate the TDC gene by directly targeting its promoter [43]. Over expression of CrWRKY in *C. roseus* hairy root induces the expression of TDC and the ZCT TFs and represses the ORCA2, ORCA3 and CrMYC2 expression [43]. In addition methyl jasmonate inducible WRKY family genes are involved in the production of defense compounds such as flavanoids and terpenoids in *Medicago- truncatula* [49].

In this report, we have used an integrated approach of transcript and metabolite analysis in opium poppy to understand the response after wounding. We have reported eighty novel expressed sequence tags (ESTs), obtained after repetitive cDNA subtractions followed by DNA array-hybridization in response to 5 hours of wound in opium poppy seedlings. One of the wound inducible EST(accession number- GT617707) was found to encode a WRKY type protein and the corresponding gene was named as PsWRKY. Characterization of PsWRKY protein led us to understand its contribution in the regulation of BIAs pathway in *Papaver somniferum*.

## Materials and Methods

### Plant Material, Stress and Hormone Treatment

Seeds of opium Poppy (*Papaver somniferum L.* cv. sampada) were germinated in soil and grown for 8 weeks at 18°C to 22°C day/10°C to 15°C night with 50% relative humidity and a photoperiod of 10 h with appropriate watering in the green house. For wound stress treatment seedling leaves were wounded with sterile pins with an average wounding site of approximately one per mm^2^ [50]. The stem has also been punctured thoroughly by sterile pins from bottom to top, avoiding any major injuries. In addition the margins of leaves were crushed with pliers. Intact seedlings were uprooted and RNA was isolated for further study. For analysis of BIA pathway alkaloids upper surface of immature capsule, straw and leaf were injured (Scratched-15 scratches/capsule, straw and leaf) using a sterilized blade. The scratched region was removed and the rest of it was used as wounded capsule, leaf and straw [32]. For methyl jasmonate treatment 50 µM solution was prepared in ethanol using 0.1% triton X-100 and sprayed over the plants. Plants treated with 0.1% triton X-100 in ethanol were taken as control. Dehydration, salt (150 mM) and ABA (100 µM) treatments were given as described in [51]. Samples were harvested after a time interval of 1, 3 and 5 hours from both treated and control plants.

### RNA Isolation, Construction of Subtracted cDNA Library, Sequencing and Sequence Analysis

Total RNA was isolated from whole seedling using TRIzol Reagent (Life Technologies, Rockville, MD). PolyA(+) RNA was purified by the oligodT cellulose method (Stratagene, Cedar Creek, TX). The subtracted CDNA library was constructed using Clon-Tech PCR-select cDNA subtraction kit (BD Biosciences Clontech Laboratories, Palo Alto, CA, USA) following the manufacturer's protocol. The subtracted and enriched DNA fragments were cloned into T/A cloning vector (pT-Adv; CLONTECH Laboratories). *Escherichia coli* DH5α were transformed with the ligation mix and plated on Luria-agar plate containing ampicillin, IPTG and X-gal for blue-white selection [52]. Plasmids were isolated according to standard alkaline lysis procedure [52]. In total, 800 clones were generated and sequenced using vector specific M13forward and M13reverse primers on an automated Applied Biosystems XL 3130 Genetic Analyzer (Applied biosystem Inc.USA) using BigDye terminator vs 3.1. Sequences were analyzed using BLAST and manually edited for the removal of vector sequences. Putative function was assigned to each expressed sequence tag (EST) on the basis of sequence similarity to proteins with known functions in the NCBI non redundant (nr) database using BLASTX. The good quality sequences were submitted to the EST data bank of NCBI and accessions obtained.

### Library Amplification, Preparation of DNA Arrays, Hybridization, and Data Analysis

Individual clones of the subtracted cDNA library were amplified using M13 forward and reverse primers. Purified PCR products of individual clones were denatured by adding an equal volume of 0.6 M sodium hydroxide. Equal volume of each denatured PCR product (about 100 ng) of more than 200 bp of size was spotted on Hybond N membranes (Amersham Pharmacia Biotech, Uppsala) in duplicate using dot-blot apparatus (Life Technologies, Bethesda, MD) in 96 well formats to make two identical arrays. Opium poppy actin cDNA (GenBank accession no. EB740770) amplified through specific primers (Table S1) was spotted as an internal control to normalize the signals of two different blots corresponding to wounded and control samples. Neomycin phosphotransferase (NPT II) gene (GenBank accession no. AF354045) was spotted as a negative control. Expression ratio was calculated according to Seki et al. 2002 [53]. Reverse northern analysis was performed as described in boominathan et al. 2004 [54].

### Real-time and Semi-Quantitative RT PCR

Candidate genes for quantitative expression analysis (qPCR) were chosen from the above reported ESTs. RNA was extracted from control as well as treated samples after 1, 3, and 5 hours of treatment. Relative quantification of 16 gene transcripts involved in BIAs pathway was analyzed after treatment with the wound and methyl jasmonate at an interval of 1, 3 and 5 hours using real time PCR and Taq Man probe-chemistry. Three independent RNA isolations were used for cDNA synthesis, and each cDNA sample was subjected to real-time PCR analysis in triplicates. Real time primers used in this study were given in Table S1.

*PsWRKY* expression analysis was performed using real-time PCR with primer pair designed from unique 3′ region having sequences WRKY_RTF- 5′-TGTTATTCGGATCGGACTGT-3′ & WRKY_RTR 5′-CCATATCATAAAACCAAGGACTTAAGG-3′. We have used eight week old seedlings to monitor the expression of *PsWRKY*. For dehydration treatment, seedlings were carefully removed from the pot and subjected to dehydration for 1, 3 and 5 hours by keeping it in between 3 MM papers (Whatman, Clifton, NJ) at room temperature. In control, seedlings were removed from the soil and immediately replanted in the same pot and kept under the same condition for the respective period of time. For cold treatment, seedlings were kept at 4° C and samples were harvested after 1, 3 and 5 hours. Seedlings kept under normal growth condition were harvested at respective time point and taken as internal control. Salt stress treatment was given by removing seedlings from the soil, and roots were dipped into aerated deionized water with or without 150° mM of NaCl. Wounding and methyl jasmonate treatments were given as described in the previous section. For ABA treatment, seedlings were removed from the soil as before, and the roots were dipped into aerated deionized water with or without 100 mM of ABA for 1, 3, and 5 hours. The control of salt and ABA treatments were removed similarly from the soil and kept in deionized water.

For semi-quantitative expression analysis, approximately 200ng of total RNA was converted to cDNA by RT-PCR (reverse transcription PCR), followed by 24 cycles of semiquantitative RT-PCR using oligo (dt) primer and Superscript II (Invitrogen) enzyme. ESTs were amplified with their respective gene specific primers mentioned in the primer list (Table S1) and thereafter analyzed on 1% agarose gel.

### Analysis of benzylisoquinoline alkaloids

High performance thin layer chromatography (HPTLC) was used for estimating the alkaloid contents in tissue samples. Tissues harvested after 5 hours of wound treatment were dried and ground to fine powder. The powdered samples were soaked overnight in 4 ml methanol and filtered through whattman No.1 filter paper. Extracts were concentrated by drying it in a water bath and re-dissolving in methanol for loading on silica gel 60F254. Standards of Morphine, thebaine, codeine and papaverine were loaded in parallel for comparison. The mobile phase used was toluene: Acetone: Methanol: Ammonia (40:40:8:2). Metabolite analysis was performed using the HPTLC system as described in [55], [56]. The data represent the mean value ± SD of two independent experiments performed in triplicates. Sanguinarine was extracted from 1g of powdered tissue to prepare acidified methanol (2.0% HCL) extract by hot percolation at 50°C for 1 hour. The extract was filtered, pooled and neutralized with ammonia solution (40%). The pH of the extract was maintained at 9.0. Finally the extract was further fractionated with diethyl ether. The fractions were pooled, concentrated under vacuum and stored at 4°C prior to chromatographic analysis. Sangunarine was estimated using high-performance liquid chromatography–photodiode array detector (HPLC–PDA-MS) system – (Shimadzu Kyoto, Japan).

### Protein Expression and Gel Mobility Shift Assay

PsWRKY protein coding sequence was amplified with gene specific primer sequences PsWRKYF1 5′-GGATCCTGGATGGGTAGTTCAAATTC-3′ and PsWRKYR1 5′-GGATCCACTAAATGTGCCTAGCTATC-3′ having BamHI sites, and cloned in pGEX4T2 in frame with GST. The sense and antisense clones were identified by sequencing of the constructs using vector specific primers PGEXF 5′-GGCAAGCCACGTTTGGTG-3′ and PGEXR 5′-GAGCTGCATGTGTCAGAGG-3′. Protein purification and Gel mobility shift assays were performed as described previously [51], [57], with 21 bp monomer sequence containing the W-box having (TTGACT/TTGACC) as a consensus cis-element. The oligos were radio-labeled with Taq-DNA ploymerase using [α-32P]-dCTP as described in [51], [57].

### Transactivation Assay of PsWRKY

PsWRKY protein was cloned and expressed in pGBKT7 in frame with GAL4 DNA-BD at NdeI-BamHI site using primer sequences PgWRKY-F 5′-CATATGTGGATGGGTAGTTCAAATTC-3′ and PgWRKY-R 5′-GGATCCACTAAATGTGCCTAGCTATC-3′. Transactivation of reporter genes were performed as described previously [51], [57].

### Promoter activation and GUS Assay

TYDC promoter was amplified with a pair of primers, TYDC-PF 5′ -GAATTCCACACCCAACTCATCATTCA-3′ and TYDC-PR 5′- GAGCTCTGATAGAGTTTGACTAAGGG-3′. The 889-bp region of TYDC promoter was cloned in pHIS vector at EcoRI and SacI restriction site. The construct was named as pyTYDC-HIS. *PsWRKY* ORF was cloned at HindIII site of the pGAD, using a primer pair PsWRKYpGF 5′-AAGCTTATGTGGATGGGTAGTTCAAATTC-3′ PsWRKYpGR 5′-AAGCTTACTAAATGTGCCTAGCTATC-3′ leading to removal of SV40 activation domain of pGAD. The resulting PsWRKY was expressing constitutively from the alcohol dehydrogenase promoter. The promoter activation assay was performed as described earlier [57].

For the reporter assay, the 889 bp 5′-UAS sequence of *TYDC* was cloned between the HindIII and NcoI site of pCAMBIA1305.1 with a pair of primer TYDC-TAF 5′-GCAAGCTTCACACCCAACTCATCATTCA-3′ and TYDC-TAR 5′-CCATGGTGATAGAGTTTGACTAAGGG-3′. For construction of effector plasmid *PsWRKY* was cloned between XbaI and BamHI site with a primer pair PsWRKY-TAF 5′-TCTAGATGGATGGGTAGTCAATTC-3′ and PsWRKY-TAR 5′-CCGGATCCTATCTTTCACGATAGCTAGG-3′ into the binary vector pBI121 which was modified by removing GUS gene after digestion with SmaI and SacI. The constructs were chemically mobilized into *Agrobacterium tumefaciens* strain GV3101. The recombinant constructs were verified by sequencing. As internal controls, empty vectors (pBI121 and pCAMBIA 1305.1) were co-transfected to normalize the GUS activity. Protoplast isolation from suspension culture of tobacco BY-2 cell lines, PEG-mediated transformation and staining for GUS activity were performed according to the techniques described by Lee et al. [58]. Fluorometric GUS assays were performed as described by Berger et al. [59]. The results are based upon three independent protoplast co-transfection experiments.

## Results

### Identification of Wound Inducible ESTs

**Figure 2:**
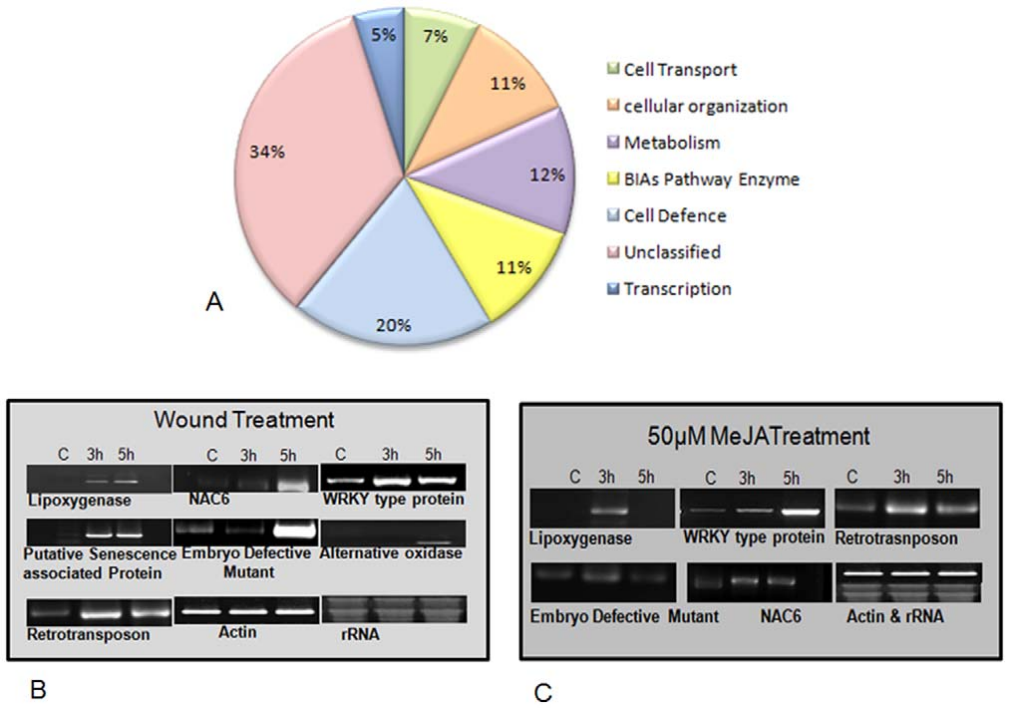
Classification and expression analysis of ESTs. (A) Functional classification of expressed sequence tags (ESTs) generated after 5 hours of wound treatment in Opium Poppy. (B) Semi-quantitative RT-PCR (reverse transcription PCR) analysis of selected ESTs, confirmed increased accumulation of transcripts after 3 and 5 hours of wounding (C). Wound induced transcripts were also monitored after 3 and 5 hours in response to exogenous application of 50 μM methyl jasmonate. Actin and rRNA were used as loading controls.

A subtracted cDNA library was constructed from poly (A+) RNA isolated from eight weeks old seedlings of wounded and control *P. somniferum*. The whole library was represented by around 1500 recombinant clones. A total of approximately 800 clones were randomly picked, stocked and sequenced using vector specific M13 forward and reverse primers. Out of 800 sequences thus generated, 167 non redundant sequences in total were found to be of good quality, and further submitted to Gen Bank (NCBI). Wound responsive ESTs were validated by hybridizing radio-labelled first strand cDNA probes, using poly (A+) RNA isolated from control and wound stressed samples. Repetitive DNA array hybridization was performed for 167 generated sequences, out of which eighty ESTs were showing more than two fold induction after 5 hours of wounding. Fold expression of ESTs was calculated according to Seki et al. [53]. Effective signal intensities of the spots were calculated after subtracting the background and normalizing it with the intensity of the negative control (NPT II). Fold induction was presented as the expression ratio (wound to control) of each ESTs to that of actin. A list of 80 unique transcripts along with their annotations, average fold inductions, e-values, and standard deviations are presented in Table 1. The representative results shown have been repeated thrice with three different sets of wounded cDNA probe to verify the reproducibility. Based on the BLASTX results ESTs (80) were classified into seven different categories such as metabolism (12.2%), transcription (4.8%), cellular transport (7.2%), cell defense (19.5%), cellular organization (11.0%), BIAs Pathway enzymes (11.0%) and unclassified (34.3%) (Figure 2A). There were a number of redundant clones suggesting their abundance in wounded samples. Most notable were NAC transcription factors (5 clones), Dirigent related proteins (8 clones), WRKY transcription factors (7 clones) etc. Wound induced transcripts were subjected to semi-quantitative and real time expression analysis to validate the differential dot-blot hybridization result. Semi-quantitative RT PCR analysis of seven randomly selected ESTs confirming the dot-blot analysis is shown in Figure 2B. In DNA-Array hybridization analysis, methyl transferase genes involved in BIAs biosynthetic pathway, namely *6-OMT* (GO238828), *7-OMT* (GO238834), and *4-OMT* (GO238826) showed maximum induction of 20.34, 12.24 and 8.32 fold respectively (Table 1). Other than BIAs pathway transcripts, Unnamed protein (GO238816) showed induction of 6.9 fold followed by induction of 7. 93 fold in dirigent related protein (GO238823).

**Table 1:** A List of total 80 ESTs obtained after repetitive round of subtractive cDNA library preparation in response to 5 hours of wounding whose fold expression was determined by nylon filter hybridization.

S.NO	Accesion number	Gen bank match	Annotation	E Value	Fold induction 5H wounding	SD(±)
1	GO238739	ABO20851	Putative senescence-associated protein	1.00E-52	4.82	1.38
2	GO238811	ABR26094	Retrotransposon protein	3.00E-28	3.89	1.26
3	GO238746	XP_002322583	aquaporin, MIP family, SIP subfamily	3.00E-54	2.94	1.05
4	GO238748	BAF02775	Alternative oxidase	6.00E-50	2.89	1.26
5	GO238751	YP_001845271	Acetyl-CoA carboxylase alpha subunit	1.00E-46	4.5	0.9
6	GO238753	NP_179763	EMBRYO DEFECTIVE 2219	8.00E-29	4.48	1.33
7	GO238755	BAH03477	Secretory carrier-associated membrane protein	2.00E-20	2.18	0.56
8	GO238759	AAX85983	NAC6 protein	2.00E-24	4.73	0.84
9	GO238761	BAF47124	Hydroproline-rich glycoprotein	1.00E-87	6.86	0.88
10	GO238762	CAQ58078	Lipoxygenase	3.00E-39	3.62	1.24
11	GO238763	AAZ32862	C43 putative splicing factor Prp8	1.00E-101	3.56	1.08
12	GO238771	CAQ43070	Putative puroindoline b protein	7.00E-30	3.49	0.65
13	GO238772	AAW26562	SJCHGC09076 protein	4.00E-04	3.3	0.8
14	GO238787	EEF29431	proteasome subunit alpha type, putative	1.00E-70	2.11	1.05
15	GO238788	ACJ04786	Late embryogenesis abundant protein2	3.00E-17	4.27	0.51
16	GO238780	YP_001714639	Putative acetolactate synthase	4.00E-87	3.2	0.4
17	GO238779	ABA29156	putative lipoic acid synthase	3.00E-24	2.67	0.68
18	GO238783	ACG38997	speckle-type POZ protein	2.00E-49	3.43	0.33
19	GO238782	ABO11871	taurine ATP-binding transport system component	1.00E-23	3.1	0.8
20	GO238736	AAA34116	Ribulose-1,5-bisphosphate carboxylase small subunit	2.00E-21	3.8	0.7
21	GO238756	BAA03104	light-harvesting chlorophyll a/b-binding protein	1.00E-115	3.9	0.5
22	GO238749	YP_001846180	GTPase, translation factor	5.00E-32	5.9	1.3
23	GO238830	AAX56303	S-norcoclaurine synthase 1	2.00E-39	8.56	1.28
24	GO238831	AAX56304	S-norcoclaurine synthase 2	3.00E-53	7.41	1.81
25	GO238834	AAQ01668	(R, S)-reticuline 7-O-methyltransferase	2.00E-105	12.24	1.36
26	GO238835	AAU20769	berberine bridge enzyme (bbe1) gene	6.00E-35	6.86	0.89
27	GO238833	ACI45390	salutaridinol 7-O-acetyltransferase	2.00E-30	22.56	2.38
28	GO238828	AAP45315	(R, S)-norcoclaurine 6-O-methyltransferase	1.00E-75	20.34	3.26
29	GO238826	AAP45313	S-adenosyl-L-methionine:3′-hydroxy-N-methylcoclaurine 4′-O-methyltransferase	4.00E-72	8.32	1.22
30	GO238827	AAP45316	S-adenosyl-L-methionine:coclaurine N-methyltransferase	3.00E-62	17.56	1.9
31	GO238832	AAC61841	tyrosine/dopa decarboxylase	1.00E-52	3.23	1.24
32	GO238741	XP_002332604	Predicted protein	6.00E-22	5.1	0.7
33	GO238812	EED14557	hypothetical protein	2.00E-17	5.6	1.7
34	GO238807	YP_001158490	chitin-binding domain-containing protein	3.00E-06	6.89	2.12
35	GO238816	CAO15875	unnamed protein product	1.00E-21	6.9	2.3
36	GO238764	CAO45611	unnamed protein product	3.00E-63	5.4	0.6
37	GO238825	ABA43635	metallothionein-like protein	1.00E-16	4.33	0.93
38	GO238822	CAN75906	Hypothetical protein	2.00E-48	4.5	0.5
39	GO238821	CAN75420	Hypothetical protein	1.00E-67	8.96	1.24
40	GO238819	AAS79670	glutamate decarboxylase 4a	6.00E-30	3.59	0.84
41	GO238818	NP_196194	DNAJ heat shock N-terminal domain-containing	3.00E-48	4.72	1.3
42	GO238775	NP_173035	Unknown protein (ARABIDOPSIS)	7.00E-12	6.3	0.8
43	GO238801	ACJ13941	Cycling DOF factor 2	1.00E-12	2.86	0.33
44	GO238838	CAO42486	unnamed protein product	6.00E-13	5.7	0.6
45	GO238843	ACL99378	hypothetical protein	5.00E-05	6.8	0.3
46	GO238840	ZP_01963577	hypothetical protein RUMOBE_01295	4.00E-33	4.4	0.9
47	GO238823	ABE73781	dirigent-related protein	4.00E-23	7.93	0.93
48	GO238842	XP_002322816	predicted protein	3.00E-22	3.9	0.7
49	GO238844	AAR98738	signal peptide peptidase	2.00E-05	4.38	1.21
50	GO238845	ABE03627	phylloplanin	6.00E-19	3.24	0.97
51	GO238847	CAA33262	unnamed protein product	7.00E-12	3.7	0.3
52	GO238824	EEF44540	hypothetical protein RCOM	8.00E-14	3.8	0.9
53	GO238820	CAO46694	unnamed protein product	6.00E-68	3.5	0.5
54	GO238803	AAM74382	Hypothetical protein	1.00E-69	3.3	86
55	GO238800	AAV44205	unknow protein	8.00E-35	4.5	0.4
56	GO238797	BAG58112	unnamed protein product	7.00E-04	3.4	0.7
57	GO238778	EEC70731	hypothetical protein	2.00E-17	4.3	0.5
58	GO238744	XP_002333400	predicted protein	2.00E-23	4.8	1.6
59	GO238816	EEF32063	sorting nexin 3	1.00E-20	8.91	1.22
60	GO238852	CAN63391	hypothetical protein	4.00E-29	3.6	0.5
61	GO238853	ABA55731	zeaxanthin epoxidase	2.00E-22	4.8	0.3
62	GO238854	EAZ13293	hypothetical protein	6.00E-29	3.6	0.4
63	GO238855	XP_001949157	similar to ribosomal protein	6.00E-22	3.48	0.5
64	GO238856	EEF40830	vacuolar ATP synthase	3.00E-12	2.58	0.77
65	GO238857	AAU03363	Wound/stress protein	2.00E-43	6.6	0.9
66	GO238858	XP_002316264	Predicted protein	1.00E-21	3.3	0.8
67	GO238859	XP_002439075	Hypothetical Protein	2.00E-23	2.9	0.5
68	GO238849	P27082	Superoxide dismutase	2.00E-40	6.6	1.8
69	GO238846	XP_2330392	Light harvesting complexII	4.00E-75	3.54	0.6
70	GO238806	XP_002521317	Putative Flavanoid 3-Hydroxylase	9.00E-61	4.4	1
71	GO238802	ACT55264	Formin2A	3.00E-04	5.2	1.2
72	GO238789	CAK18872	hypothetical protein	3.00E-22	5.7	1.4
73	GO238737	XP_002521686	Putative NAC domain containing protein	5.00E-22	3.9	0.5
74	GT617707	AAP85545	WRKY type DNA binding protein	8.00E-19	8.6	1.1
75	GT617708	XP_002275053	Hypothetical protein	6.00E-08	6.3	4.4
76	GT617713	XP_002311200	Predicted protein	1.00E-06	3.9	0.6
77	GT617718	ACJ84895	unknow protein	4.00E-11	2.5	0.5
78	GT617724	ZP_01252775	Thiamin biosynthesis lipoprotein	2.00E-08	2.9	0.6
79	GT617725	XP_002269352	Similar to KEA1	8.00E-35	4.8	0.3
80	GT617726	AAK57516	ACC oxidase	3.00E-39	5.2	0.7

The data presented means of duplicate experimental repeats with ±SD values. Fold induction of some of the transcripts was further analyzed with semi-quantitative expression analysis.

### Response of Wound inducible Transcripts to Wounding and Exogenous Application of MEJA

Semi-quantitative RT-PCR (Reverse transcription PCR) analysis of randomly selected seven ESTs namely ‘lipoxygenase, (GO238762)’ ‘Alternative oxidase (GO238748)’, ‘Embryo defective mutant (GO238753)’, ‘Retrotrasposon (GO238811)’, ‘NAC6 transcription factor (GO238759)’, ‘Putative senescence associated protein (GO238739)’ and ‘WRKY type protein (GT617707)’ had shown more than 2-fold induction after wounding (Figure 2B), supporting the DNA array hybridization results. The central role of jasmonic acid in plant responses to wounding is well established and jasmonic acid-dependent and -independent wound signal transduction pathways have been identified [60]. Therefore, we analyzed the expression of above mentioned wound induced ESTs in response to exogenous application of 50μM methyl jasmonate (Figure 2C). Lipoxygenase, NAC6, retrotransposon, Embryo defective mutant and WRKY transcripts showed induced expression after wounding as well as exogenous application of methyl jasmonate treatment (Figure 2C).

### Real Time Expression Analysis of Genes Involved in Alkaloid Biosynthetic Pathway

**Figure 3:**
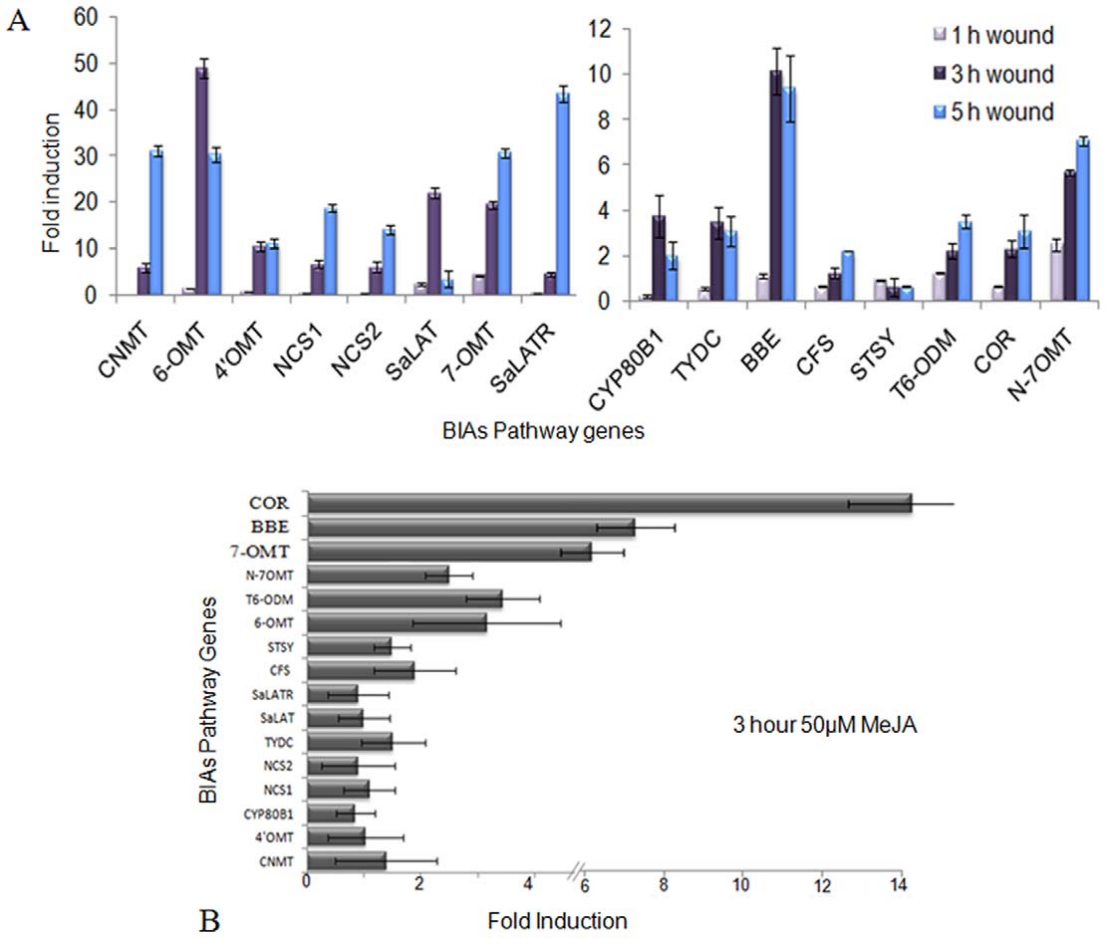
Real Time expression analysis of selected BIAs biosynthetic pathway genes. (A) The expression analysis of transcript level of BIAs biosynthetic pathway genes after 1, 3, and 5 hours of wounding. Intact seedlings without wounding were taken as control at respective time points. (B) Real time expression analysis of BIAs pathway genes after 3 hours of exogenous application of 50 μM methyl jasmonate. Intact seedlings treated with an equal volume of ethanol in 0.1% triton X-100 was taken as control. The data represented means of triplicate biological and experimental repeats, error bars represented SDs.

DNA macroarray expression analysis and functional classification of ESTs suggested that a major category of ESTs induced in response to wound belong to ‘metabolism’, which includes BIAs biosynthetic pathway genes. We have analyzed the expression of 16 gene transcripts of BIAs pathway after 1, 3, and 5 hours of wounding in seedlings using real time and taq-man probe chemistry. Transcripts of *SALAT* and *7OMT* were induced early after one hour of wounding (Figure 3A). Three hours of wounding induced *6OMT* up to 40 fold, followed by *SALAT* and *7OMT* (Figure 3A). Transcript level of *CNMT, 6OMT, 4-OMT, NCS1, NCS2, 7OMT, CYP80B1* and *SALAT reductase*, were induced more than 10 fold even after 5 hours of wounding. *COR* transcript did not induce in response to wound (Figure 3A). Exogenous application of methyl jasmonate induced *7OMT, 6OMT, N7OMT, BBE* and *COR* transcripts. The induction of *COR* was up to 10 fold after 3 hours of methyl jasmonate treatment (Figure 3B). *BBE, 7OMT, 6OMT, N7OMT* and *T6ODM* transcripts were induced both in response to wounding as well as methyl jasmonate treatment in intact seedlings. Cheilanthifoline (CFS) and Stylopine synthase (STSY) did not show any significant change in their transcript level after wounding (Figure 3A).

### Analysis of benzylisoquinoline alkaloids in Response to Wounding

**Figure 4:**
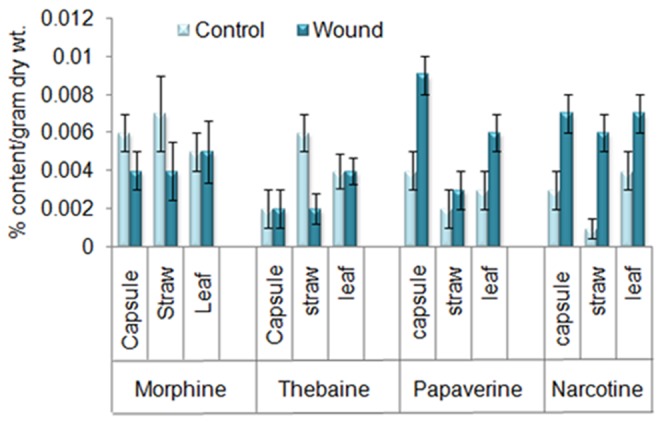
Analysis of benzylisoquinoline alkaloids. Morphine, narcotine, papaverine and thebaine were analyzed after 5 hours of wounding in different tissues (straw, capsule, and leaf) of Opium Poppy. The data represented in % content/gram of dry weight. Data was an average of two independent quantifications repeated in triplicate and error bars represented SDs.

Tissue specific metabolite analysis was performed in response to wounding in 130 day old immature plants. We analyzed morphine, narcotine, papaverine, thebaine, codeine and in leaf, straw and capsule in response to five hours of wounding and sanguinarine was estimated in capsule, straw and root. The level of morphine in capsule, straw and leaf decreased after wounding. The reduction in level of morphine in straw was 42.8% in comparison to control condition. In wounded capsule morphine level went down by 33.3% in comparison to control capsule (Figure 4). The level of narcotine and papaverine showed increased accumulation after wounding. In comparison to control tissue papaverine showed maximum accumulation in capsule (125%) followed by leaf (100%) and straw (50%) (Figure 4). On the other hand increased level of Narcotine was found maximum in straw (500%) followed by capsule (133%) and leaf (75%) (Figure 4). Thebaine level showed differential accumulation in different tissues, its level decreased in straw (66.6%) whereas in leaf and capsule no significant difference was obtained (Figure 4). Codeine level did not show any significant difference after wounding. The level of sanguinarine was estimated using HPLC and was not found to have much significant difference after wounding (Figure S1).

### *PsWRKY* encodes a WRKY family protein

**Figure 5:**
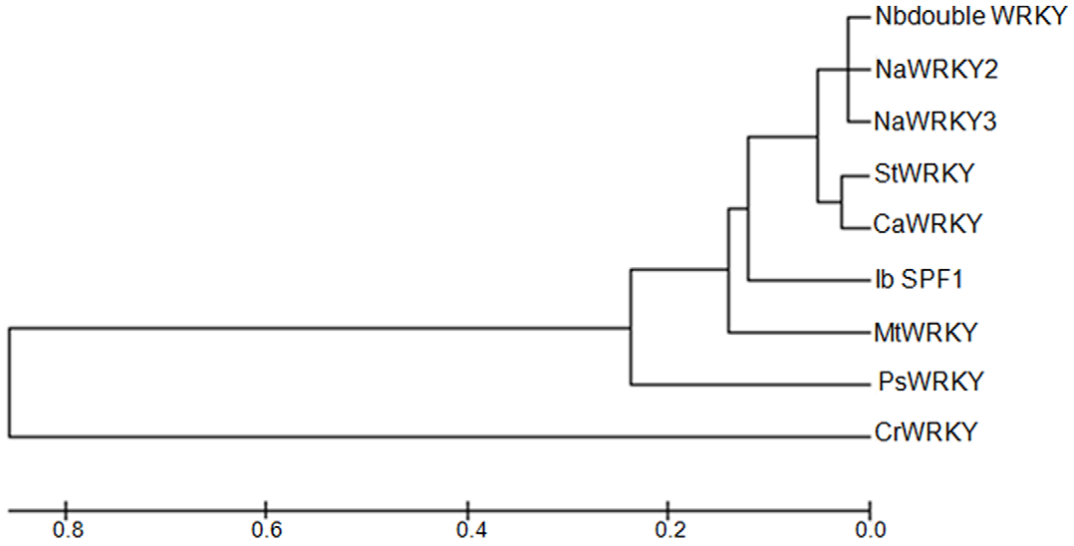
Phylogenetic analysis of PsWRKY. phylogenetic tree of PsWRKY and selected WRKY family proteins from other plant species was constructed by Neighbor joining method using MEGA5 software. The statistical reliability assessed by bootstrap value provided along with the tree. Selected WRKY proteins, respective plant species and GenBank accession numbers are as follows: Nbdouble WRKY [Nicotiana benthamiana double WRKY, Accession no. BAI63296.1], NtWRKY2 [Nicotiana tabacum, Accession no. BAA77383.1], NaWRKY3 [Nicotiana attenuata, Accession no. AAS13439.1], CaWRKY [Capsicum annuum, Accession no. ABD65255.1], StWRKY [Solanum tuberosum, Accession no. BAI63294.1], SPF1 protein [Ipomoea batatas, Accession no. ABD65255.1], MtWRKY [Medicago trucatula, Accession no. XP_003615949.1], CrWRKY1 [Catharanthus roseus, Accession no. ADT82685.1].

Identified wound induced EST (accession number- GT617707) showing homology with WRKY type proteins, was made full length using 5′ as well 3′ RACE with a primer pair WRKY5′RF- 5′-GTAACTACCCCTAGCAGCAG-3′ and WRKY3′RF 5′-CTGCTAGGGGTAGTTACAGA-3′. A sequence of 1797-bp size was obtained having a conserved WRKY domain in the deduced amino acid sequence and was named *Papaver somniferum* WRKY (PsWRKY; GenBank accession no. JQ775582). *PsWRKY* was having 1,107-bp open reading frame encoding a predicted protein of 369 amino acids. It was encoding a conserved double WRKY DNA binding domain of 56 and 59 amino acids. Identified *PsWRKY* was also containing conserved WRKYGQK residues required to interact with the major groove of DNA having conserved 6 bp (TTGACC/T) residues (Figure S2). Phylogenetic analysis of *PsWRKY* showed that PsWRKY and CrWRKY (Catharanthus WRKY) shared the same lineage in a cluster, having Medicago WRKY and SPF1 from Sweet potato as other closely related WRKY proteins (Figure 5).

### Real Time Expression of PsWRKY

**Figure 6:**
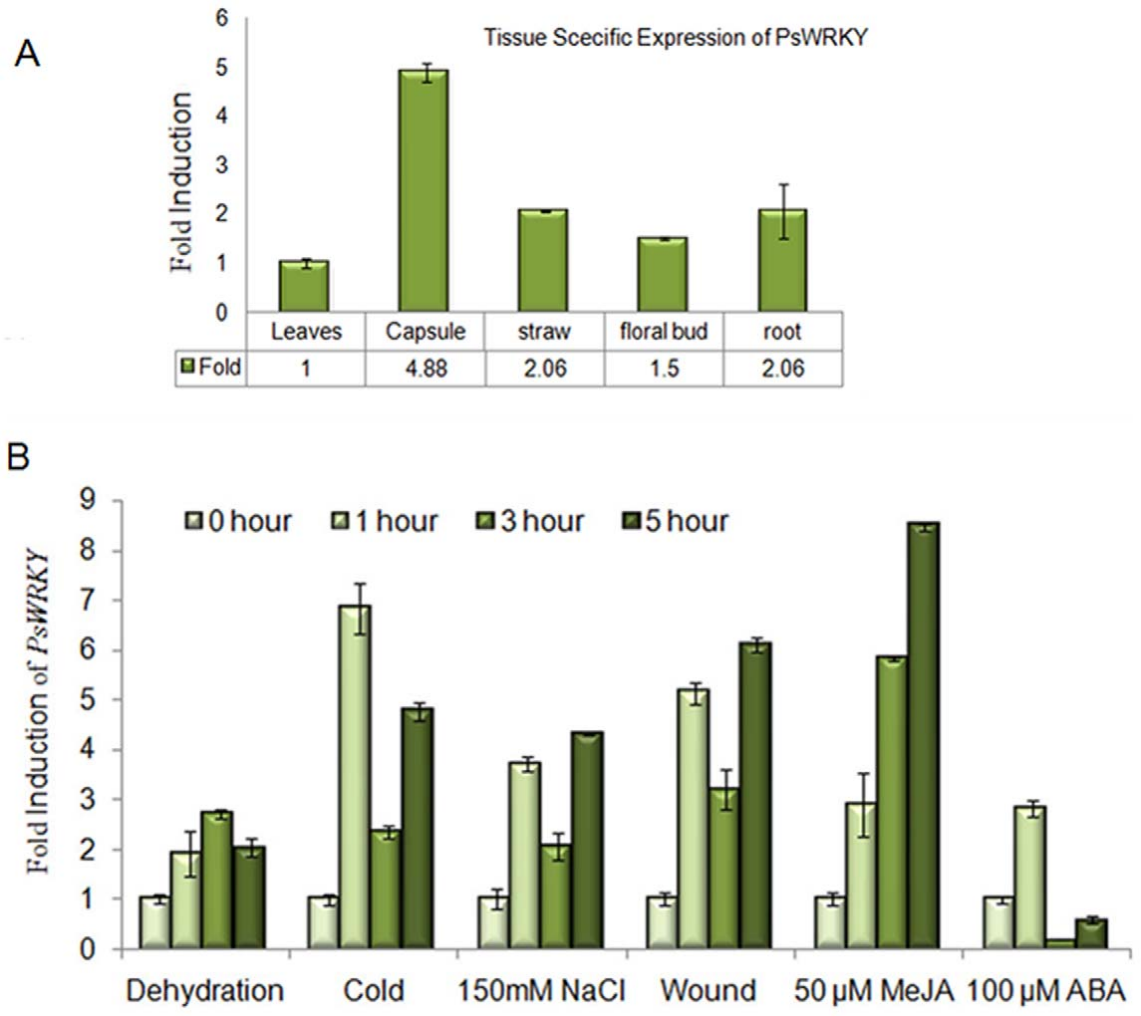
Real time expression analysis of *PsWRKY* under control and different stress conditions in Opium poppy.

Real time expression analysis was used to study the expression of PsWRKY transcript under different stress conditions. Real-time primers were made from a PsWRKY CDNA fragment representing the unique C-terminal end of the protein. Very low basal level of PsWRKY transcript was detected under control condition showing its requirement in normal development or metabolic processes. The maximum expression of PsWRKY under control condition was observed in capsule, followed by straw and root, which are the major sites of metabolite synthesis and accumulation in opium poppy (Figure 6A). Under dehydration transcript level of PsWRKY showed maximum induction of 2.5 fold after 3 hours and then decrease in its level after 5 hours (Figure 6B). Its transcript level reached a maximum of more than 6 fold after 1 hour and decreased after 3 hours in case of cold treatment (Figure 6B). Salt stress induced PsWRKY transcript after 1 hour of treatment; then went down after 3 hours and again induced maximally after 5 hours (Figure 6B). Accumulation of PsWRKY in response to wounding showed early induction i.e. just after 1 hour of treatment, its transcript level decreased to 2.5 fold after 3 hours, however it showed a maximum induction of 6 fold after 5 hours (Figure 6B). Methyl jasmonate treatment also induced PsWRKY transcript in a time dependent manner (Figure 6B). After ABA treatment PsWRKY transcript showed maximum accumulation of 2 fold just after 1 hour and it went down to basal level at 3 hours and 5 hours.

### PSWRKY Protein Activates Transcription in Yeast and binds *in vitro* to W-box element

**Figure 7:**
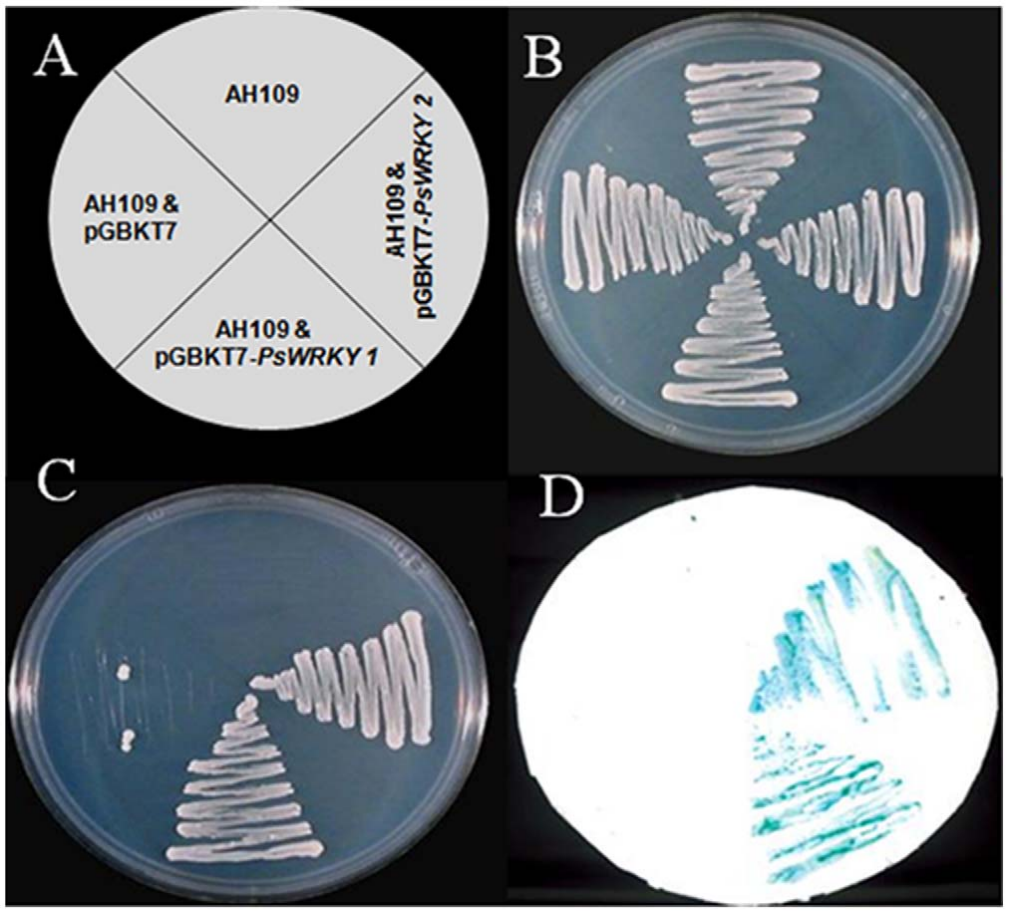
Transactivation of reporter genes in yeast by PsWRKY protein. *PsWRKY* cDNA cloned in yeast expression vector (pGBKT7) fused with GAL4 DNA BD, was transformed into yeast strain carrying three reporter genes, *HIS3*, *ADE2*, and *LacZ*, under the control of the *GAL4* promoter. (A) Growth of Yeast colonies carrying no vector (Con), vector only (Vec), and two transformants (PsWRKY-1 and PsWRKY-2) having *WRKY* on YPDA (B) and on synthetic medium lacking His and Ade (C). Activation of the third reporter gene as in (D) was shown by β-galactosidase assay of the transformants using ONPG.

Transactivation analysis of PsWRKY protein was demonstrated using yeast (*Saccharomyces cerevisiae*) one hybrid assay. *PsWRKY* ORF was cloned at NdeI and EcoRI sites of the pGBKT7 vector (CLONTECH) to express the protein in fusion to GAL4 DNA-BD. The resulting construct was transformed into AH109 carrying *HIS3*, *ADE2* and *LacZ* reporter genes under *GAL4* promoter. BD-PsWRKY transformed colonies were selected on medium lacking His and Ade. Strain AH109, vector transformed AH109, along with two BD-PsWRKY clones were able to grow on YPDA medium (Figure 7B). On the other hand only BD-PsWRKY1 and BD-PSWRKY2 colonies having PsWRKY were able to grow in medium lacking *HIS3*, *ADE2* (Figure 7C). PsWRKY transformed colonies also showed β-galactosidase activity in ONPG assay (Figure 7D).

**Figure 8:**
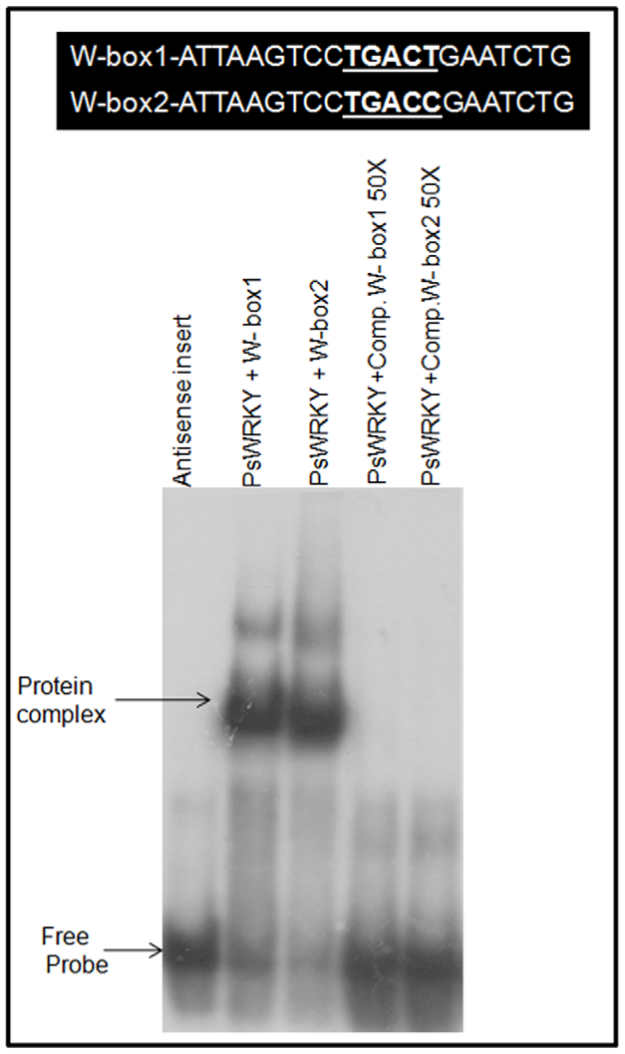
Gel-retardation assay demonstrating that PsWRKY protein binds to the W-box probe. The probes (1 ng) used in all reactions were ^32^P-labeled dimers of the oligonucleotides shown in W-box1 and W-box2. The two consensus *cis*-motifs with sequence TGACT and TGACC predominantly identified in BIAs pathway gene promoters were used in the oligonucleotide. Recombinant PsWRKY protein expressed in *E. coli* Bl21 (DE3) in fusion with GST was purified with GST-agarose columns. PsWRKY cloned in antisense orientation was also purified and used as a negative control.

The gel retardation assay was performed to demonstrate that PsWRKY protein binds specifically to W-box (TTGACT/C) a conserved consensus DNA binding motif present in the identified and cloned promoters of BIAs pathway genes in *P. somniferum*. PsWRKY ORF was cloned in pGEX4T-2 vector (Amersham) and expressed in *E. coli* BL21 (DE3) to produce PSWRKY protein fused with glutathione-s-transferase at its N-terminus. Bacterially expressed GST-PsWRKY showed gel shift when run with a probe containing W-box motif having TTGACT (W-box1) or TTGACC (W-box2) oligo-nucleotide sequence in it, while a GST-fused antisense clone of PsWRKY did not. The gel shift produced was competed out with an excess of cold probe (50X) showing that GST-PsWRKY protein specifically binds with the W-box (Figure 8).

### Activation of TYDC promoter

**Figure 9:**
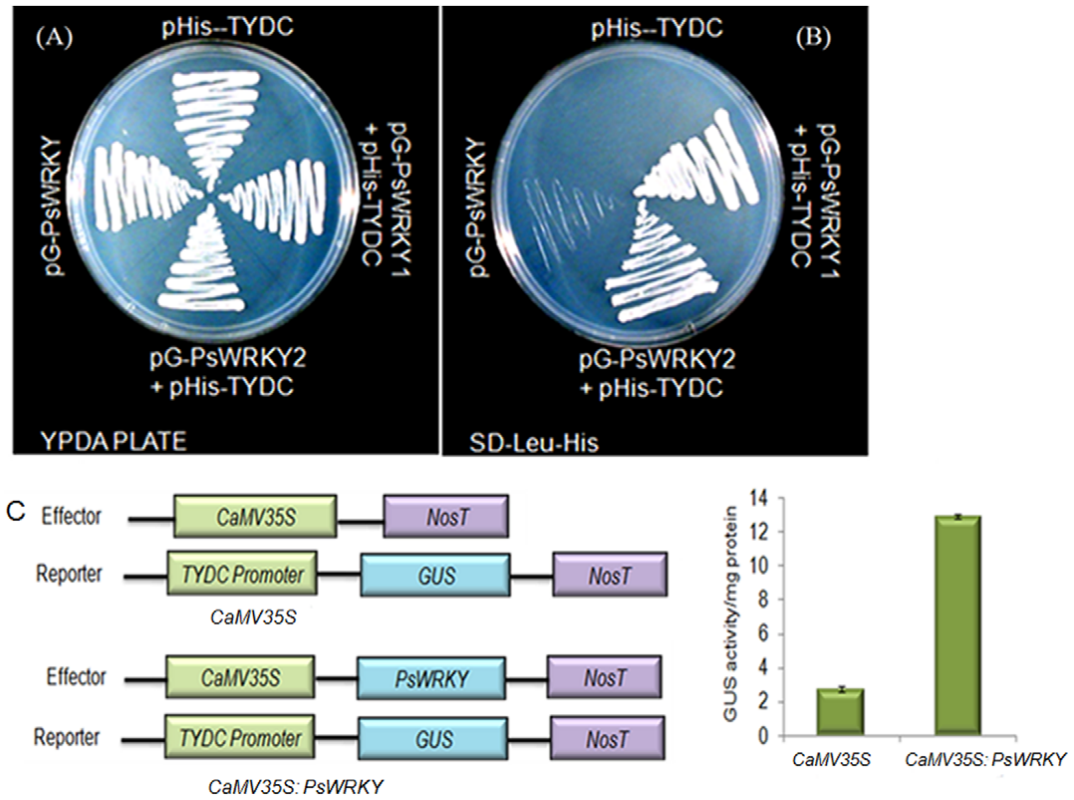
Activation of TYDC promoter by PsWRKY protein in yeast *S cerevisiae* AH109 (HIS^−^LEU^−^) and tobacco BY2 Cells. *PsWRKY* cDNA cloned with LEU marker under a constitutive alcohol dehydrogenase promoter was transformed. The resulting transformants were further transformed with TYDC promoter harboring HIS marker. (A) The transfrormants were grown at 30°C on YPD and (B) synthetic drop out medium lacking the histidine and leucine. Two individual transformants carrying PsWRKY are shown. (C) Activity of GUS reporter construct fused to *tydc* 5′-upstream activating sequence in tobacco BY2 cells harboring empty vector or PsWRKY. The promoter–GUS construct was introduced through *Agrobacterium* into the tobacco BY2 cells already transformed with control vector or PsWRKY. GUS activity level in the PsWRKY-expressing protoplasts relative to that in the control BY2 cells is presented. The data presented means of triplicate transformation repeats and error bars represented SDs.

To understand the molecular basis of differential expression of BIAs biosynthetic genes in response to wounding, we analyzed the known promoter sequences of five transcripts namely *4OMT, 7OMT, SAT, BBE* and *TYDC*. Upstream regions of these transcripts were screened for *cis*-acting regulatory elements using software e.g. PLACE, *CIS*TER or plant care database [61]. Wound responsive *cis*-regulatory elements i.e. W-box, was found out to be conserved in all five of them with a conserved motif of TTGACY (Y = T/C) binding site (Table S2). We have cloned the tydc promoter region of 889-bp in front of the auxotropic selection marker HIS in the plasmid pHIS2.1 (ClonTech, Palo Alto, CA, USA). This construct was co-introduced with another plasmid (modified pGAD with a LEU selection marker [57]), having *PsWRKY* cDNA cloned under the constitutive yeast *alcohol dehydrogenase (Adh1)* promoter in the His- Leu- strain AH109 of *S. cerevisiae* (Clontech). The transformants were selected on HIS-LEU- medium (Figure 9). Only *PsWRKY* cDNA or *tydc* promoter region was also transformed and streaked on HIS-LEU- medium (Figure 9). The transformants having PsWRKY and *tydc* were able to grow on YPDA and HIS^−^LEU^−^ medium (Figure 9A). On the other hand transformants lacking either *PsWRKY* cDNA or *tydc* promoter region were not able to grow on selection medium lacking His & Leu suggesting that PsWRKY was able to activate *tydc* promoter (Figure 9B).

To further investigate the effect of PsWRKY on *tydc* expression, we cloned the 889-bp-long 5′-upstream activating sequence (UAS) of *tydc* (GenBank accession number: AF025434). Sequence analysis identified three potential W-box -binding elements (TGACY) similar to the WRKY TFs binding site, at 521 (+), 855 (−) and 756 (+) bp upstream of the translational start site of *tydc* [13]. Upstream activating sequence of 889 bp, including the 285 bp 5′-UTR of *tydc*, was fused to the *GUS* reporter gene by replacing the CaMV35S promoter of pCAMBIA1305.1. The construct was mobilized into the vector control and PsWRKY-expressing tobacco BY2 cells through *Agrobacterium*-mediated transformation. The transformants were selected on 30 µg/ml of hygromycin and 50 µg/ml of kanamycin. Transformed BY2 cells were assayed for GUS activity. GUS activity in the PsWRKY-expressing BY2 protoplast cells was more than 13-fold than that in the control vector transformed BY2 cells, showing that PsWRKY can activate transcription from the *tydc*-5′-UAS (Figure 9C).

## Discussion

**Figure 10:**
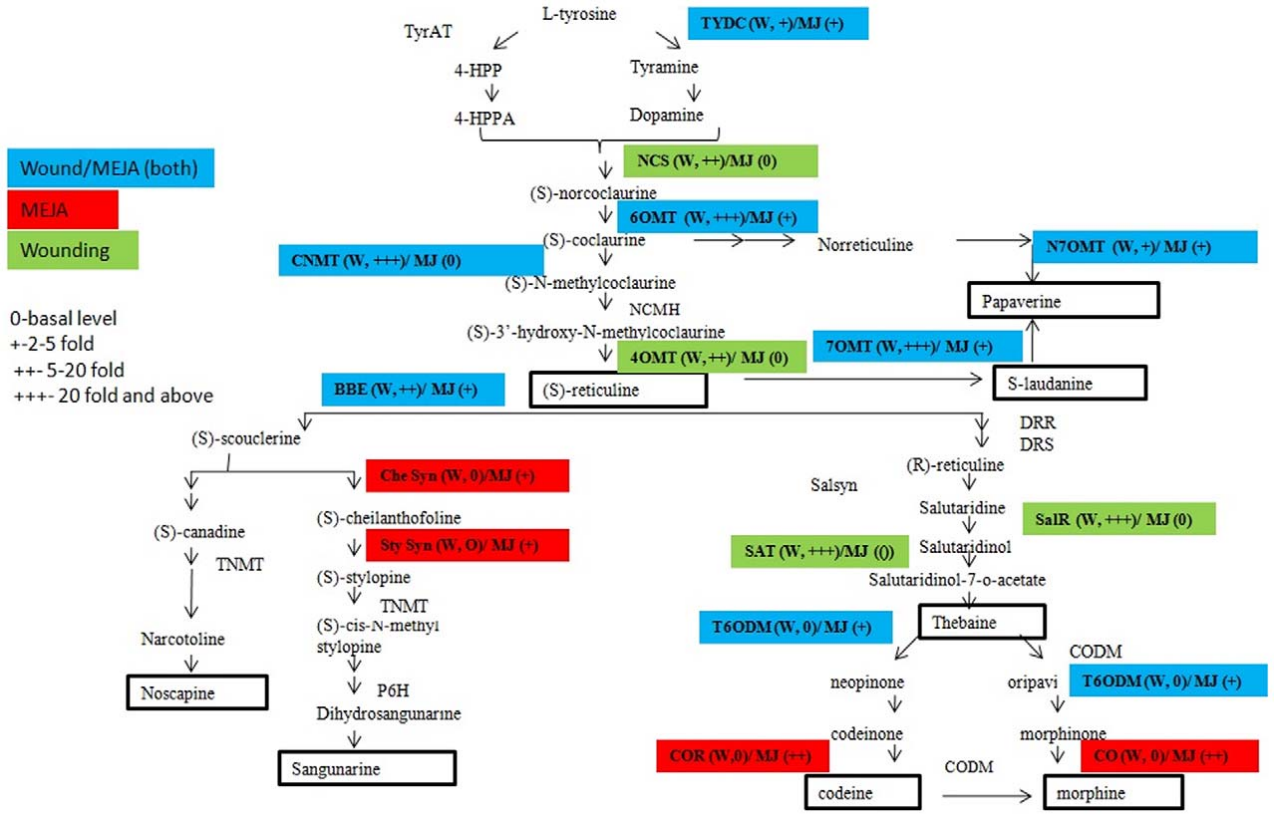
Biosynthetic pathways showing the expression of BIA transcripts in *Papaver somniferum* after wounding and methyl jasmonate treatment. Enzymes shown in blue are commonly induced by both wounding as well as methyl jasmonate. Enzymes shown in red are induced by methyl jasmonate only and enzymes in green are induced by wounding only. Fold induction was represented in comparison to actin.

We have selected opium poppy for this study because wounding is done on the walls of the green seed pod, for the exudation of latex. Isolated dry latex is used for a number of pharmacological purposes [1]. Opium poppy cell cultures are treated with elicitors, which induces the sanguinarine biosynthetic pathway leading to increased sanguinarine accumulation in cell cultures. ESTs identified after elicitor treatment in cell cultures were having 40 enzymes connecting sanguinarine biosynthesis to sucrose catabolism. Other than sanguinarine biosynthetic pathway ESTs, most of the elicitor study in cell cultures has identified induced expression of morphinan pathway transcript SAT1 [7]. Real time expression analysis of 16 gene transcripts of BIAs pathway showed that 13 gene transcripts namely *TYDC*, *CNMT*, *6OMT*, *4-OMT*, *NCS1*, *NCS2*, *7OMT*, *BBE*, *SalR*, *CYP80B1*, *T6ODM*, *SAT* and *N7OMT,* were induced after wounding (Fig. 3A). Out of which seven namely *TYDC*, *6OMT*, *CNMT*, *CYP80B1*, *4-OMT*, *BBE*, *SAT* were found out to be commonly induced after wounding in seedling and cell suspension culture studies [34]. Novel transcripts of BIAs pathway *7OMT*, *SalR*, *T6ODM* and N7OMT were showing induced expression in response to wound in seedlings in comparison to cell cultures. Maximum induction after wounding in seedlings was obtained for BIAs transcripts *6-OMT*, *7OMT*, *SalR*, *N7OMT*, *BBE*, and *SAT*. Although *BBE* showed induced expression, transcripts of *CFS* and *STSY* involved in sanguinarine biosynthesis were not induced after wounding. Induced expression of *BBE* might regulate increased accumulation of narcotine instead of sangunarine. Induced expression of *7OMT* and *N7OMT* transcripts further supported the increased accumulation of papavareine. Thus BIAs alkaloid analysis after wounding in plants correlates with their transcript expression data. Representation of BIAs pathway, its transcript expression after wounding and methyl jasmonate are shown in Figure 10. Exogenous application of methyl jasmonate induced only *6OMT*, *7OMT*, *BBE*, *COR* and *T6ODM* transcripts in seedling (Figure 3B). The possible differences identified in the accumulation of BIAs transcripts after wounding and methyl jasmonate treatment in seedlings and cell culture might be due to the lack of de-differentiated cell cultures to synthesize and store morphine and its pathway intermediates [35].

Wounding up-regulates the secondary metabolic pathways involved in activation of defense response [62]. Wound induced expressions of MYB transcription factors regulate the expression of flavonoid genes [62]. Wound responsive genes identified in this study were classified according to functional attributes such as cell defense, cellular localization, metabolism, BIAs pathway enzymes, transcription and unclassified. The most abundant group was represented by an unclassified group of ESTs, showing that many transcripts are still unexplored in opium poppy and can be a useful target for further studies. Identification of novel abiotic and biotic stress responsive genes like ‘Aquaporin (GO238746)’, ‘LEA (GO238788)’ and ‘Glutamate decarboxylase (GO238819)’ (Table 1) and their induced expression may relate to the increased denovo synthesis of ABA after wounding [63]. Wounding in opium poppy seedlings induced some of the novel enzymes associated with secondary metabolic pathways. A homologue of dirigent protein (GO238823) catalyzing the stereochemistry of compounds and involved in lignan biosynthesis, was identified in wounded seedlings showing induction up to 7.9 fold. A novel ‘Lipoxygenase homologue (GO238762)’ showed induced expression after wounding as well as methyl jasmonate treatment. Wounding in opium poppy has also identified three novel transcription factors, including WRKY domain containing transcription factor (GT617707) & two different ‘NAC domain transcription factors (GO238759), (GO238737) showing induced expression of more than two fold. Identification of consensus wound inducible W-box motif and up- regulation of specific metabolites along with their transcripts prompted us to explore the possibility of identifying WRKY as a regulator of BIAs pathway. Phylogenetic analysis of PsWRKY with other selected WRKY proteins showed maximum percentage identity of 51% with CrWRKY (*Catharanthus roseus*) and MtWRKY (*Medicago truncatula*) followed by 47% with SPF1 (*Ipomea batatas*). Both *CrWRKY* & *PsWRKY* are upregulated in response to methyl jasmonate [43]. But unlike *CrWRKY*, *PsWRKY* showed induced expression in response to cold, dehydration, wounding, and salt treatment. *PsWRKY* expresses mainly in capsule, followed by straw and root while CrWRKY expresses mainly in root followed by fruit and leaves. Transcript expression study suggested that PsWRKY and CrWRKY may have different roles and are regulated by different or additional pathways.

Recombinant PsWRKY was able to bind specifically with W-box element present in the promoter region of BIAs transcripts. Yeast one hybrid assay and protoplast transient analysis have demonstrated that PsWRKY was able to activate the tydc 5′UAS confirming the direct involvement of PsWRKY in the regulation BIAs biosynthetic pathway. However in vivo functional study of PsWRKY will further provide a more complete understanding of its role in regulation of BIAs pathway.

## Conclusion

The study has identified eighty novel wound inducible transcripts in *Papaver somniferum*. Integrated transcript and metabolite profiling of benzylisoquinoline alkaloids revealed that wounding increased the accumulation of narcotine and papaverine in opium poppy. Novel wound inducible PsWRKY protein interacts with the W-box, consensus *cis*-element present in BIAs pathway gene promoter and activates transcription from TYDC promoter.

## Supporting Information

Figure S1 **Detection of Sanguinarine.** Sanguinarine was analyzed in control and wounded tissue samples using HPLC. No significant amount of sanguinarine was detected as represented in chromatograms.
(DOC)

Figure S2 **A deduced cDNA and amino acid sequence of PsWRKY cloned from** ***Papaver somniferum*** **.** WRKY domains are indicated by underline.
(DOC)

Table S1 **List of Primers used in this study.**
(DOC)

Table S2 **List of WBOX elements in the promoters of the BIAs pathway genes.** Summary of putative cis regulatory wound responsive elements in known promoters of BIAs pathway genes.
(DOC)
