# Supplementary material for: Wound Induced Tanscriptional Regulation of Benzylisoquinoline Pathway and Characterization of Wound Inducible PsWRKY Transcription Factor from Papaver somniferum
Source: PLoS One. 2013 Jan 30;8(1):e52784. doi: 10.1371/journal.pone.0052784 (PMC3559656; doi:10.1371/journal.pone.0052784)

Figure S1. **Detection of Sanguinarine**: Sanguinarine was analysed in control and wounded tissue samples using HPLC. No significant amount of sanguinarine was detected as represented in chromatograms

Analytical conditions:

Column: C18 (4.6x250mm, 5µm)

Mobile: A-1.0%AcOH

B- Acetonitrile

Gradient:

TIME %B

0.01 20

15 95

20 20

25 stop

Flow-1.0ml/min

Det-327nm

ESI- [M+H]+ 332

Std- 0.2mg/ml

Sample= Prepared as described in Materials and Method

Results: Not detected in all six samples.

STD-Sanguinarine


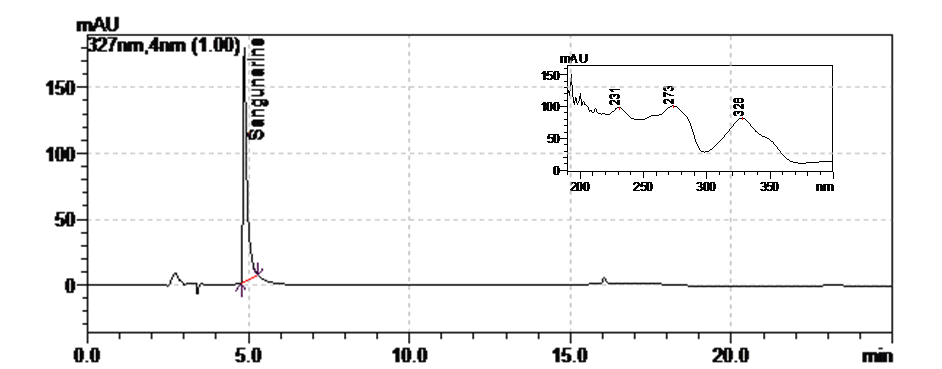


Cotrol root


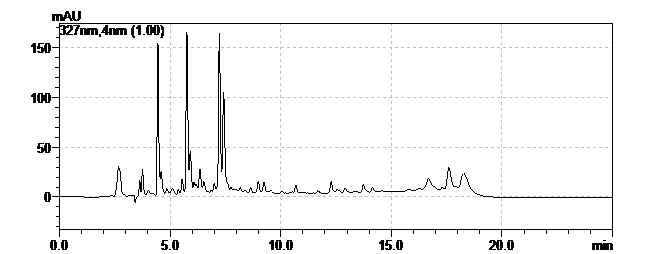


Wounded root


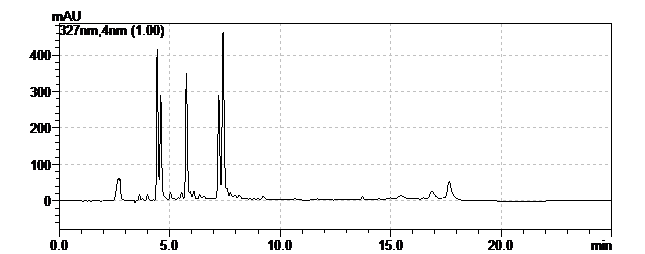


Control straw


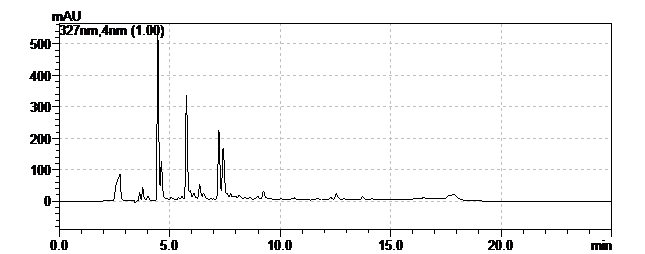


Wounded straw


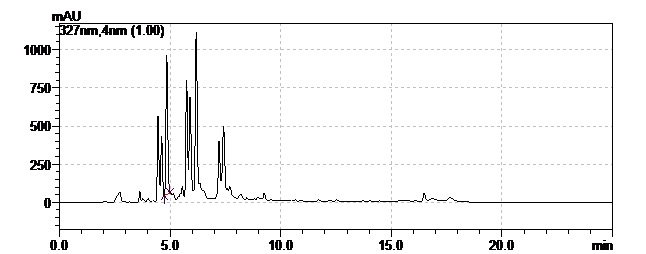


Control capsule


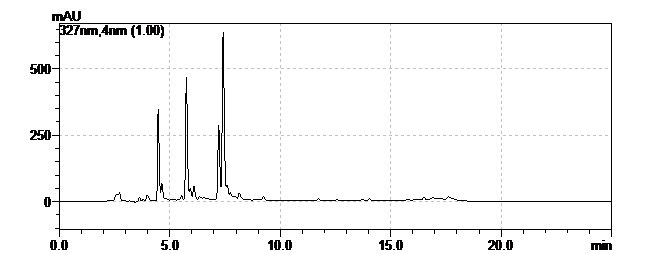


Wounded capsule


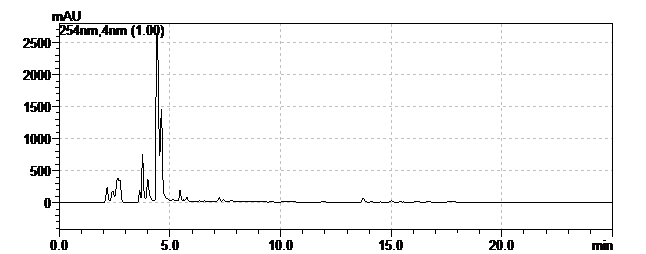

Supplement: Figure S1 — Detection of Sanguinarine. Sanguinarine was analyzed in control and wounded tissue samples using HPLC. No significant amount of sanguinarine was detected as represented in chromatograms. (DOC) [file pone.0052784.s001.doc]
