# Supplementary material for: Wound Induced Tanscriptional Regulation of Benzylisoquinoline Pathway and Characterization of Wound Inducible PsWRKY Transcription Factor from Papaver somniferum
Source: PLoS One. 2013 Jan 30;8(1):e52784. doi: 10.1371/journal.pone.0052784 (PMC3559656; doi:10.1371/journal.pone.0052784)

Figure S2. **A deduced cDNA and amino acid sequence of PsWRKY**: A cDNA and amino acid sequence of PsWRKY cloned from *Papaver somniferum*. WRKY domains are indicated by underline.


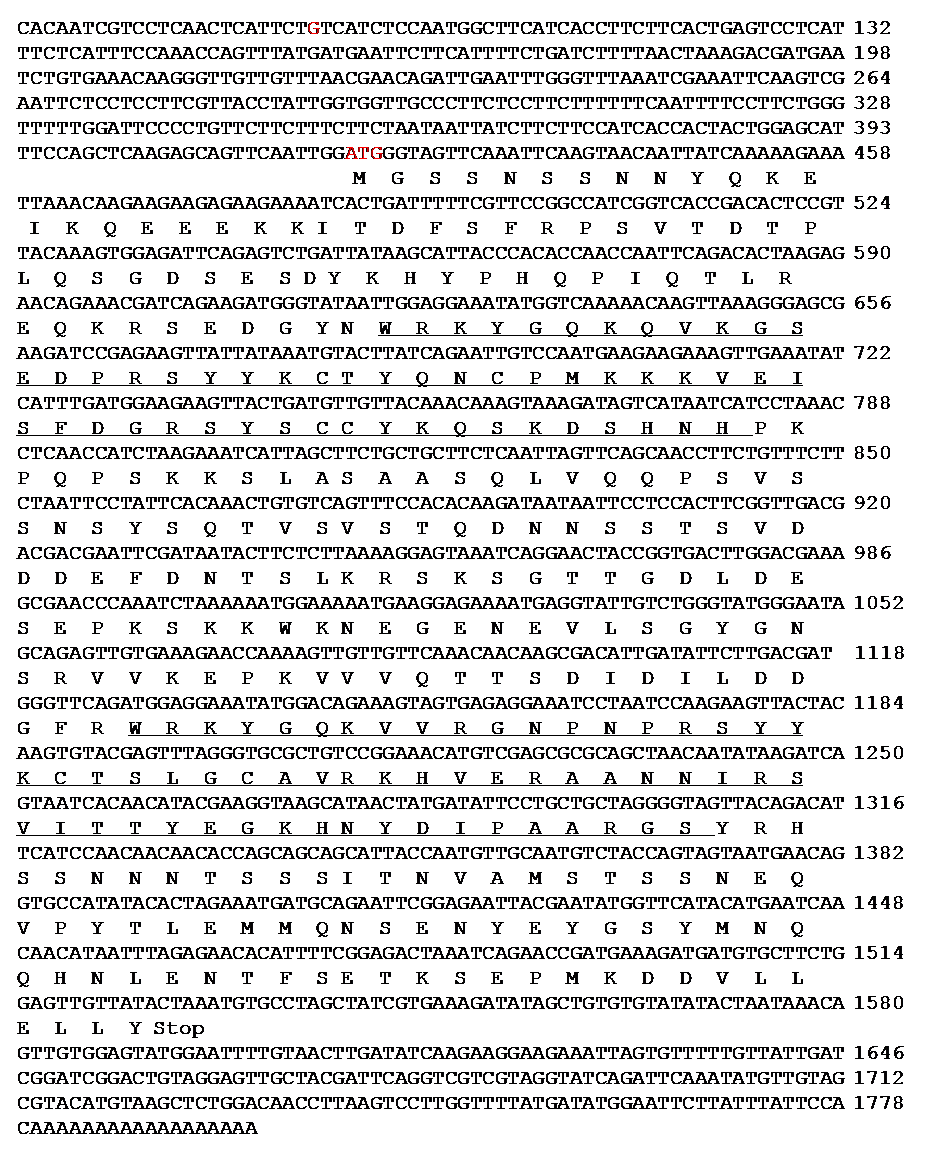

Supplement: Figure S2 — A deduced cDNA and amino acid sequence of PsWRKY cloned from Papaver somniferum . WRKY domains are indicated by underline. (DOC) [file pone.0052784.s002.doc]
