# Supplementary material for: Wound Induced Tanscriptional Regulation of Benzylisoquinoline Pathway and Characterization of Wound Inducible PsWRKY Transcription Factor from Papaver somniferum
Source: PLoS One. 2013 Jan 30;8(1):e52784. doi: 10.1371/journal.pone.0052784 (PMC3559656; doi:10.1371/journal.pone.0052784)
Supplement: Table S1 — List of Primers used in this study. (DOC) [file pone.0052784.s003.doc]

Table S1. List of Primers used in this study

| Lipoxygenase | LIPF: ATTGGGAAAAATGACGATGGAAAA  LIPR:CTACGTTATCCCTTGTAAACCATTC |
| --- | --- |
| Alternative oxidase | AOF: CAATGCCTATTTCTTATCTTACATG  AOR:GATCTGAGCCAAGTATTCAATATAA |
| Cytochrome P450-TBP | CYPF: GAGCTCATCTTAGGACACCTGCG  CYPR: CGCAATGTGATTTCTGCCCAGTG |
| NAC transcription factor | NACF:CTACAACGGCAGAGCACCACGGG  NACR: TGGCTAAGTTATAGTAGTAAGTTG |
| Retrotransposon | RTRF: TCGGGACCAACAAGGGGTAGTACAG  RTRR:TTCATCGTCGAGAGGGAAACAGCC |
| Putative Senescence associated protein | PSAF:AGACATTGTCAGGTGGGGAG  PSAR:ATCTAGGTTGAATTACTACGC |
| WRKY Protein | WRKYF:GATTGATGGGTATAATTGGAGG  WRKYR:CTAGTGATTTGGGCGTGTTTG |
| Embryo defective(EMB) | EMBF: TACTTGAGGAGAACCATGGTACGG  EMBR: ACTGTCAACTTGAGATTCCTCTG |
| Actin F:  Actin R: | PACTINF: AGAGAGATTCCGTTGTCCAGAGG  PACTINR: GACCGGAACTCATCGTATTCTCC |
| T6ODMF  T6ODMR | AAAACAAGGAAGCTCGATGGAA  GCACCAAAAAACGACAAAAGG |
| STSY F  STSY R | TGCGAAATCGTGAAAGTTGAA  TGTCTCTACAAGCTTTATGTGGTATAGCT |
| CHF FS  CHF RS | AGTTCATGCCCGAGAGATTCC  TCCATTCCAGCACAGATTCG |
| N7OMTF  N7OMTR | TTCTGTGATTCGTCTTGCTTCTAATT  CCTTTATATTCATTCCACATTGTGATATG |
