# Supplementary material for: Wound Induced Tanscriptional Regulation of Benzylisoquinoline Pathway and Characterization of Wound Inducible PsWRKY Transcription Factor from Papaver somniferum
Source: PLoS One. 2013 Jan 30;8(1):e52784. doi: 10.1371/journal.pone.0052784 (PMC3559656; doi:10.1371/journal.pone.0052784)
Supplement: Table S2 — List of WBOX elements in the promoters of the BIAs pathway genes. Summary of putative cis regulatory wound responsive elements in known promoters of BIAs pathway genes. (DOC) [file pone.0052784.s004.doc]

| Gene | Sequence | Start Position | Putative Motif | Reference |
| --- | --- | --- | --- | --- |
| S-adenosyl-L-methionine:hydroxy-N-methylcoclaurine transferase | TTGAC  TGACT  CTGACY  TGACY | 95(+),545(+), 40( ),129(-), 314 (-)314(-)  96(+)  223 (+)  96(+), 224(+), 128(-) , 360(-)  96 (+) | WBOXATNPR1  WBOXHVISO1  WBOXNTCHN48  WBOXNTERF3 | Yu *et al*., 2001  Sun *et al.,* 2003    Yamamoto et al., 2004  Nishiuchi *et al.,* 2004 |
| salutaridinol acetyltransferase (SAT) gene | TGACY | 278 (-) | WBOXNTERF3 | Nishiuchi *et* *al.,*2004 |
| somniferum reticuline-O-methyltransferase (7OMT) gene | TGACT  TGACY | 140 (+), 357 (+), 440 (+)  140(+), 367(+), 440(+) | WBOXHVISO1  WBOXNTERF3 | Sun *et al.,*2003  Nishiuchi *et* *al.,*2004 |
| Berberine bridge enzyme (bbe1) gene | TTGAC  TGACT  TGACY | 186(+),638(+), 663(-)  639 (+)  639 (+) | WBOXATNPR1  WBOXHVISO1  WBOXNTERF3 | Yu *et al.,*2001  Chen *et al.,* 2002  Nishiuchi *et* *al.,*2004 |
| tyrosine/dopa decarboxylase gene | TGACT  TGACY | 521 (+), 855 (-)  521 (+), 756 (+), 855 (-) | WBOXHVISO1  WBOXNTERF3 | Sun *et al.,*2003  Nishiuchi *et* *al.,*2004 |

Table S2. **List of WBOX elements in the promoters of the BIAs pathway genes**: Summary of putative cis-regulatory wound responsive elements in known promoters of BIAs pathway genes.
